# Changes in the Upper and Lower Pharyngeal Airway Spaces Associated with Rapid Maxillary Expansion

**DOI:** 10.5402/2012/290964

**Published:** 2012-06-18

**Authors:** Fitin Aloufi, Charles B. Preston, Khalid H. Zawawi

**Affiliations:** ^1^Division of Orthodontics and Periodontics, Dental Department, Security Forces Hospital, Riyadh Colleges of Dentistry & Pharmacy, P.O. Box 84891, Riyadh 11681, Saudi Arabia; ^2^Department of Orthodontics, School of Dental Medicine, State University of New York at Buffalo, Buffalo, NY 14214-3008, USA; ^3^Division of Orthodontics, Department of Preventive Dental Science, Faculty of Dentistry, King Abdulaziz University, P.O. Box 80209, Jeddah 22254, Saudi Arabia

## Abstract

*Objectives*. The primary objectives of this retrospective study were first to compare the upper and lower pharyngeal airway spaces between orthodontic patients with and without maxillary constriction and second to evaluate the effect of rapid maxillary expansion (RME) on these airway spaces. A secondary objective was to compare the mode of breathing between groups. *Materials and Methods*. The experimental (RME) group consisted of 30 patients (mean age, 14.2 ± 1.3 years, 16 boys and 14 girls) with maxillary constriction who were treated with hyrax-type RME. The control group comprised the records of age- and gender matched patients (mean age, 13.8 ± 1.5 years, 16 boys and 14 girls) with no maxillary constriction but requiring nonextraction comprehensive orthodontic treatment. Cephalometric measurements in the sagittal dimension of upper and lower airway spaces for the initial and final records were recorded. Mode of breathing and length of treatment were also compared. *Results*. The sagittal dimension of the upper airway increased significantly in the RME group (mean = 1.3 mm) compared to the control group (mean = 0.5 mm), *P* = 0.016. However, there was no significant difference in the lower pharyngeal airway measurement between the RME group (mean = 0.2) and the control group (mean = 0.4), *P* = 0.30. There was no significant difference with respect to mode of breathing between the two groups (*P* = 0.79). *Conclusion*. Rapid maxillary expansion (RME) during orthodontic treatment may have a positive effect on the upper pharyngeal airway, with no significant change on the lower pharyngeal airway.

## 1. Introduction

Maxillary constriction is associated with several problems that include cross bite (dental and/or skeletal), occlusal disharmony, esthetics and functional problems such as narrowing of the pharyngeal airway [1, 2]. Several studies have shown that maxillary constriction may play a role in the etiology of obstructive sleep apnea (OSA) [3–5]. OSA is a condition characterized by the episodic cessation of breathing during sleep. An examination of the causes of apnea has produced several classifications for this condition. Apnea secondary to sleep-induced obstruction of the upper airway and combined with simultaneous respiratory efforts is the most common type and has been classified as obstructive sleep apnea syndrome (OSAS). OSAS results in oxygen desaturation and arousal from sleep, thus bringing about a constellation of signs and symptoms related to oxygen desaturation and sleep fragmentation. The reduced blood oxygen saturation may give rise to hypertension, cardiac arrhythmia, nocturnal angina, and myocardial ischemia. Furthermore, impaired sleep quality leads to excessive daytime sleepiness, deterioration of memory and judgment, altered personality, and reduced concentration [6, 7]. 

Several studies reported that patients with OSA have abnormal cephalometric dentofacial morphologies [1, 2, 7–10]. Also tendencies toward retrognathia, [11, 12]. micrognathia, long face and inferior positioning of the hyoid bone [13]. In addition, other features were also reported, such as: tendencies toward reduced cranial base length and angle, large ANB angle, hyperdivergent mandibular plane, elongated maxillary and mandibular teeth, narrowing of the upper airway, long and large soft palate, and large tongue [9, 10]. 

Rapid maxillary expansion (RME) is used in subjects with transverse maxillary deficiencies [14, 15]. The RME method was first introduced in 1860 and a great deal of research has been carried out since then [16]. In these studies, it was noted that RME causes not only dentofacial changes, but also craniofacial structural changes [15, 17, 18]. Furthermore, studies showed that maxillary expansion increases the volume of the nasal cavity, increases the nasal cavity width, lowers the palatal vault, straights the nasal septum, and reduces the nasal airflow resistance, hence, improves the nasal respiration [18–23]. It has been reported that RME results in a mean increase of 4.1 mm in the nasal cavity width [14]. 

Therefore, the primary objectives of this retrospective study were twofold, first to compare the upper and lower pharyngeal airway spaces between orthodontic patients with and without maxillary constriction and second to evaluate the effect of RME on these pharyngeal airway spaces. A secondary objective was to compare the mode of breathing between the groups.

## 2. Materials and Methods 

In this retrospective study, records of 30 orthodontic patients (mean age, 14.2 ± 1.3 years, 16 boys and 14 girls) who had maxillary deficiency of intermolar width less than 32 mm [24] and requiring maxillary expansion were selected. The records of 30 age- and gender-matched patients (mean age, 13.8 ± 1.5 years, 16 boys and 14 girls) with maxillary intermolar width of at least 32 mm or more and not requiring maxillary expansion but underwent nonextraction orthodontic treatment were selected as controls. The study was reviewed and approved by the Health Institutional Review Board of the University at Buffalo.

Inclusion criteria for both groups were as follows: (1) subjects between the ages 11–16 years, (2) patient who did not received previous orthodontic treatment, (3) good quality initial and final records, (4) patient received fixed orthodontic treatment (nonextraction) for at least 18 months duration as a part of the orthodontic treatment, (5) none of the patients were subjected to a surgical procedure directed at their nasal cavities or pharyngeal airway (tonsillectomy adenoidectomy) prior to or during treatment, and (6) none of the patients were diagnosed with any craniofacial disorder.

Patients in the Expansion (RME) group had a hyrax-type maxillary expander banded on the maxillary first premolars and first molars. The patients were monitored weekly for appropriate activation of the appliance. Hyrax was turned 1 or 2 times per day (0.25–0.5 mm) until the required expansion was achieved, that is, slight overcorrection of the crossbite (average time, 4–6 weeks), and then was stabilized. The hyrax was used for retention for at least 3 months after-expansion. Most patients with RME had no orthodontic treatment until after the fixed retention period. The control group started orthodontic treatment within six months of the expansion group. Cephalometric radiographs were taken of all patients as part of both initial orthodontic treatment records and final records (the day of removing the fixed orthodontic appliance therapy). The lateral cephalometric images for each subject were taken using the same imaging device. All subjects were positioned in the cephalostat with the ear rods placed in the external auditory meatus to stabilize the head with the sagittal plane at right angle to the path of the X-ray and Frankfort plane parallel to the horizon, the nose rest piece position on soft tissue nasion, the teeth in centric occlusion, and lips in relaxed and closed position.

**Figure 1:**
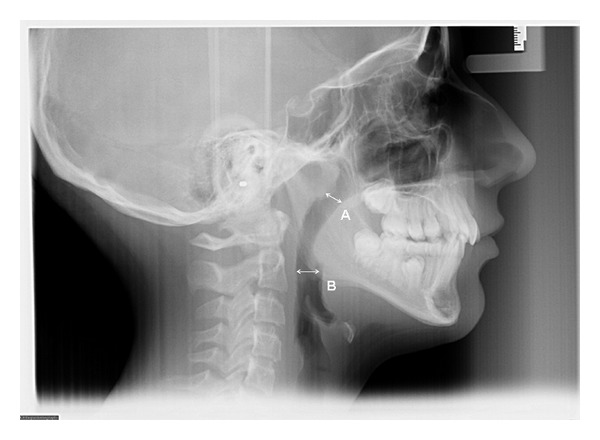
Measurements of the upper (A) and lower (B) pharyngeal airway widths were taken using tracing of the lateral cephalometric radiograph.

The dimensions of the upper and lower pharyngeal airways were measured directly from the cephalometric radiograph using a plastic ruler (Ormco) according the McNamara Airway Analysis [25]. Briefly, the upper pharynx greater depth in the sagittal dimension was measured from a point on the posterior outline of the soft palate to the closest point on the pharyngeal wall (Figure 1(A)).

The lower pharynx greater depth in the sagittal dimension was measured from a point at intersection of the posterior border of the tongue with the inferior border of the mandible to the closest point on the posterior pharyngeal wall. When double views of the mandibular inferior border were present, intersection of the views was used. (Figure 1(B)). As part of initial orthodontic records questionnaire, the mode of breathing (nasal or mouth breathing) was always recorded by asking the patients or their parents/guardians. This subjective assessment was also recorded at the end of orthodontic treatment. 

An analysis was done using the Student's independent samples *t*-test for comparison of the continuous variables (age, length of treatment, the initial airway measurements, and the changes in the airway measurements). The Chi-square contingency table was used for the categorical variables (gender and mode of breathing). All statistical tests were calculated at the 5% level of significance (*α* = 0.05). The statistical Package for the Social Science version 11.0 (SPSS, Chicago, IL) was used. 

## 3. Results

Summary of age and gender distribution for both groups is presented in Table 1. There was no significant difference between the groups at the beginning of the study with regards to age. There was an average of 4.4 months longer treatment time for the RME group compared to the control group. This mean difference was significant, *P* < 0.0001.

There was no significant difference between groups for the upper and lower pharyngeal airway measurement prior to treatment (Table 1). 

After treatment the upper pharyngeal airway was significantly increased in the RME group (mean = 1.3 mm) compared to the control group (mean = 0.5 mm). However, there was no significant difference in the lower pharyngeal airway measurement between the RME group (mean = 0.2) and the control group (mean = 0.4). When comparing the mode of breathing between groups at the beginning of the treatment, Chi-square showed that there was no significant difference. When comparing the mode of breathing from initial to end of treatment for both groups independently, 11 subjects in RME group showed improvement in the mode of breathing from mouth to nasal compared to 10 subject in the control group. However, Chi-square showed that this change was not significant (Table 2).

## 4. Discussion

Abnormalities in the craniofacial region have been recognized as part of the pathophysiology of OSA and considered predisposing factors to OSA by its adverse effects on the oropharyngeal airway. The more frequently recognized craniofacial abnormalities include narrowed posterior air space, elongation of the soft palate, mandibular deficiency, and inferiorly placed hyoid bone relative to the mandibular plane [1, 2, 26, 27]. Furthermore, maxillary constriction might play a role in the pathophysiology of OSA because maxillary constriction is associated with low tongue posture that could result in oropharynx airway narrowing, which is a risk factor for OSA [1, 2, 28]. 

In the present study, the expansion group had a significant increase in the upper pharyngeal airway, while there was no difference between the two groups in regards to the lower pharyngeal airway. Rapid maxillary expansion has been reported to be associated with an increase in nasal cavity width and significant reduction in the nasal airway resistance [18, 29]. Therefore, maxillary expansion may have a positive effect on decreasing nasal resistance and increasing the upper airway. This conclusion is in agreement with previous studies that found that RMA is an advantageous procedure in the treatment of cases with inadequate nasal capacity and exhibiting chronic nasal respiratory problems[18, 23]. In the current study, subjective evaluation showed that patients who were mouth breathers believed that their nasal breathing had improved following RME; however, they were only 37% compared to 33% for the control group (Table 2). This finding is not consistent with previous reports [30]. It appears that RME may lessen the nasal resistance even with the difficulty in demonstrating the linear changes in the size of nasal cavities [31]. It should be noted that maxillary expansion is not justified solely for improving the airway without the presence of a transverse constriction or narrowness of the maxilla [31]. It is also imperative to note that by measuring the pharyngeal airway on a lateral cephalometric radiograph, one is measuring a three-dimensional object on a two-dimensional image. However, the aim of the present paper was only to study the sagittal dimension of the airway.

There was an almost identical distribution of mouth breathers within the two groups studied with no statistically significant difference in the composition of both groups. Mouth breathing, in this study, was not associated with narrow pharyngeal airway initially and even though 11 subjects in the RME group reported improvement in the mode of breathing, compared to 10 subjects in the control group; however this was not statistically significant. This could be due to the small sample size in both groups. In a study by Warren et al. [20, 21, 32], it was found that 97% of subjects with transversal nasal area less than 0.4 cm^2^ tended to be mouth breathers to some extent and almost 12% of subjects with seemingly adequate airways were habitual mouth breathers. Mouth breathing is associated with a reduction of the retropalatal and retroglossal areas as well as lengthening of the pharynx. Mouth breathing is associated with abnormal development of the facial skeleton and occlusion and resulted in changes in the forces of the craniofacial lateral, buccal, and lingual muscles. Also, mouth breathing may cause facial abnormalities which also undermines general health [33]. 

The relative size of the nasopharynx as a cause of mouth breathing has been cited. Linder-Aronson and Leighton's comprehensive investigation of 162 mouth breathing children serves as the bench-mark study on the anatomic and physiologic features of the nasopharynx [34]. In their report the size of the nasopharynx and adenoids were related to history of a mouth breathing as well as to the measurement of nasal airflow. They concluded that adenoids lead to mouth breathing primarily in children with anatomically small nasopharynx.

The duration of treatment, in the present study, for the RME group was an average of 4.4 months longer than the control group. However, the age distribution was similar for both groups. This was important factor to consider in an attempt to control for the effect of growth. Growth as a contributing factor has been reported to change the size and shape of the nasopharynx. It is believed that the total depth of the nasopharynx is established in the first or second year of life [35], while its length continue to increase until maturity. This increase in length was attributed to the descent of the hard palate and cervical vertebrae away from the cranial base[36]. Bergland found a thirty-eight percent increase in nasopharyngeal height from six years of age to maturity [37]. 

It was found in the present study that afterm treatment, the upper and lower airways were increased in the RME and the control groups. However, the increase was only significant in the upper airway of the RME group. This finding could be explained by a recent study of 120 lateral cephalometric radiographs that were divided into three stages according to the dental age. Results from that study showed that the upper pharyngeal depth increases with age, whereas the lower pharyngeal depth was established early in life [38]. Johnston and Richardson [39] studied a sample of 16 adults who had cephalometric films taken and repeated after an interval of 32 years. They studied the changes in the pharyngeal skeletal size, pharyngeal soft tissue thickness, pharyngeal airway depth, and soft palate dimensions in addition to standard craniofacial measurements. The results showed an increase in maxillary prominence and upper and lower anterior face height. However, the nasopharyngeal skeletal dimensions were unchanged, while the anteroposterior depth of the nasopharyngeal lumen increased as a result of a reduction in thickness of the posterior nasopharyngeal wall. In the oropharynx, the depth of the airway was found to decrease with age, while the soft palate became longer and thicker. Their findings indicate that pharyngeal morphology is not immutably established during childhood and adolescence, but changes throughout adult life [39]. Future studies could be designed to study the amount of expansion and changes in pharyngeal airway, the relation of mouth breathing, depth of the upper airway, and skeletal pattern, both sagittally and vertically. Finally, a three dimensional reading using advanced digital imaging techniques could provide researchers with a better understanding of the dynamic changes in the upper and lower airways throughout orthodontic treatment.

## 5. Conclusion

A retrospective study was performed in order to assess whether maxillary expansion as part of orthodontic treatment, increases pharyngeal airway patency in orthodontic patients the following conclusions could be made

- RME group showed a significant increase in the upper pharyngeal airway space compared to the control group.
- No significant changes were observed in the lower pharyngeal spaces for both groups.
- RME did not significantly improve the mode of breathing.

Therefore, it may be concluded, within the limitation of the study, that maxillary expansion during orthodontic treatment could have a positive effect on the upper pharyngeal airway, with no significant change on the lower airway and mode of breathing. 

## Figures and Tables

**Table 1:** Comparison between experimental and control groups at initial and final records.

Parameters	RME Group (*n *= 30)	Control Group (*n *= 30)	*P*-value
Age (years)	14.2 ± 1.3	13.8 ± 1. 5	0.28^∗^
Gender	16 B, 14 G	16 B, 14 G	1.0^∗^
Treatment duration (months)	30.2 ± 3.2	25.8 ± 3.3	<0.0001
Initial upper pharyngeal airway (millimeter)	14.6 ± 1.9	15.4 ± 1.5	0.08^∗^
Initial lower pharyngeal airway (millimeter)	10.7 ± 1.5	11.3 ± 1.6	0.12^∗^
Difference (pre/post) upper pharyngeal airway (millimeter)	1.3 ± 1.1	0.5 ± 1.3	0.016
Difference (pre/post) lower pharyngeal airway (millimeter)	0.2 ± 1.0	0.4 ± 0.9	0.30^∗^

B: boys, G: girls, ^∗^Not Significant (*P* > 0.05).

**Table 2:** Distribution of subjects according to improvement of mode of breathing.

	Improved		Percent		*P* value
	Yes	No	Yes	No	
RME group	11	19	37%	63%	0.79^∗^
Control group	10	20	33%	67%	
Total	21	39			

^∗^Not Significant (*P* > 0.05).
